# Artificial Weathering of Contact-Charred Wood—The Effect of Modification Duration, Wood Species and Material Density

**DOI:** 10.3390/ma15113951

**Published:** 2022-06-01

**Authors:** Maija Kymäläinen, Tinh Sjökvist, Jakub Dömény, Lauri Rautkari

**Affiliations:** 1 Department of Bioproducts and Biosystems, Aalto University, P.O. Box 16300, FI-00076 Aalto, Finland; jakub.domeny@mendelu.cz (J.D.); lauri.rautkari@aalto.fi (L.R.); 2 Department of Forestry and Wood Technology, Faculty of Technology, Linnæus University, SE-351 95 Växjö, Sweden; tinh.sjokvist@sodra.com; 3 Department of Wood Science and Technology, Faculty of Forestry and Wood Technology, Mendel University in Brno, Zemědělská 3, 613 00 Brno, Czech Republic

**Keywords:** artificial weathering, carbonization, contact charring, surface modification, wood, wood modification

## Abstract

A relevant issue with charred exteriors is the inconsistency of the result, which makes service life predictions complicated. Contact charring enables the creation of a very evenly modified surface with accurate control of temperature and modification time, but the weathering properties are questionable. This paper evaluated the effect of the modification time relative to char layer and transition zone thickness, wood species and material density in an artificial weathering test. The results revealed higher color stability in connection to longer modification time, but also an increase in the cracked surface area. Cracking was heavily dependent on the modification regime and increased with increasing char and transition zone thicknesses. Dense spruce had the highest color stability with the most severe modification regime, but char layer thickness varied more than on other wood types. Furthermore, species-dependent cracking patterns affected the final result as the small-scale flaking experienced by birch increased the washing off of char. It is likely an even higher modification temperature with a shorter modification time is needed to produce sufficient weathering resistance suitable for exterior uses.

## 1. Introduction

Although naturally weathered wooden façades are common in some areas, it is recommendable to apply a coating to reduce wear and photodegradation of the surface and improve the service life of the claddings. Uncoated structures develop a ‘driftwood’ appearance with uneven degradation, discoloration from the sun, and surface mold, and sometimes even rot in areas of constant water stress. This sort of wear leads to ‘aesthetic instability’ that necessitates replacement even if protective functions still remain [1]. Grüll et al. [2,3] postulated that once cracking occurs, a limit state of the coated element is reached, and maintenance is required. As a hygroscopic material, wood is subject to continuous stresses exerted by cyclic humidity conditions that cause swelling and contraction. The dimensional changes are a challenge for coated wood as only a few polymeric coatings can withstand the recurring cycles [4]. Fatigue during extended weather exposure degrades the coating, and prolonged exposure often contributes to brittleness and crack formations [3]. The cracks tend to collect dirt and water, both in the form of condensed moisture from the surrounding atmosphere and raindrops. Organic material such as pollen combined with suitable moisture promotes fungal growth, while the cracks provide easy access for the hyphae to the interior parts of the wood. Alkyd and oil paints are susceptible to cracking alongside wood as they embrittle with exterior exposure. On the other hand, acrylic (latex) paints have good extensibility that allows them to withstand repetitive dimensional changes [5]. The downside is that they have high vapor permeability but release the penetrated moisture slowly [3,6]. This may lead to trapping of moisture, peeling of the coating layer and biodeterioration if sufficient ventilation is not considered. Wood density affects weathering by decreasing the erosion rate [7,8]. Sjökvist et al. [9] noted, however, that in spruce claddings, this effect was limited to an intact coating. The formation of cracks reduced the impact of density and leveled moisture content differences between high- and low-density spruce.

One-sided surface charring has recently gained wider attention as a natural, organic modification method. Because the wood is modified only on one side, it is comparable to a coating, one that is formed from the wood substrate itself. Flame-charred wood (comparable to the ancient *yakisugi* method) exhibits an extensively cracked “alligator skin” surface, where the wood is reduced to a carbonaceous char. The surface is rather inert to photodegradation [10], is also sooty and is almost impossible to clean without damage. Therefore, manufacturers often recommend coating, which brings about maintenance needs and interferes with the natural essence of the product. Contact charring is basically a similar method to flame charring in that it leaves the wood with a hydrophobic char layer, but the modification temperature can be set precisely, and repeatability is excellent. In the process, the wood is pressed against a heated surface for a given modification time. The process is not commercialized, but recent laboratory-scale studies have given insights into improved sorption properties and wettability characteristics [11,12,13,14] and changes in chemical composition and functional groups toward a more stable material [13,15,16]. Mechanical performance was found to improve in terms of modulus of rupture [13], but dimensional stability in moisture exposure was not promising [10,12,16]. The char layer depth has a great influence on the weatherability of the surface [10,13,17]. Char layer thickness can be increased by increasing the modification time, but concomitant char cracking may change the properties in terms of surface stability when in use. Cracks, in turn, have an impact on overall performance and durability in the use of wood in exterior settings. The service life of modified wood is best evaluated at the setting of intended use, but natural weathering experiments are very time-consuming. An artificial weathering setup overlooks factors such as pollutants and microbial degradation [18] but also eliminates differences between exposure sites. Some features may be different, e.g., Grüll et al. [19] found the development of cracking to be faster in artificial weathering compared to natural weathering. This was mainly due to less natural cracking during cold winters when moisture stresses are small.

As the density, as well as wood species, affects the response to charring, as well as the erosion rate of wood exposed to weathering, it is of interest to quantify these characteristics. Additionally, to date, there are no experiments describing these effects on contact-charred woods. The aim of this study is to clarify how modification time, and, consequently, the char depth, affects weathering properties of contact-charred wood. Because wood density influences the cracking behavior, as well as charring rate/char depth, two densities of spruce were used. Additionally, birch was compared with spruce wood since soft- and hardwoods exhibit different charring rates/char depths, as well as surface functionalities. This study forms part of a series that investigates the potential of charred exteriors as an option for traditional coatings, as well as modifying less-used wood species such as birch to become more suitable for exterior uses.

## 2. Materials and Methods

Norway spruce (*Picea abies* L.) and Silver birch (*Betula pendula* Roth.) were sourced from Southern Finland, from a local sawmill. To compare the effect of density, we used light and dense spruce (average densities from several samples presented in Table 1). Only sapwood from mature trees was used for these experiments. Samples with dimensions of 100 mm × 100 mm × 18 mm were cut from 2–3 boards that had been planed on the pith side and end-matched to reduce between-samples variation. Samples were conditioned at 65%RH, 20 °C. The samples were sorted into four groups per wood type with 4 samples in each. Each group corresponded to a different modification regime: unmodified reference, short (10 min), intermediate (30 min) and long (60 min) modification. The samples were coded as wood species/type (B = birch, LS = light spruce, DS = dense spruce) followed by modification time (R = reference, 10…60 = 10…60 min modification; Table 1). A steady modification temperature of 320 °C was used. The device was a stainless steel hot plate with accurate temperature control. Samples were set on the plate pith side down and a weight of 16 kg was applied on top (giving a surface pressure of 0.016 N/mm^2^). The temperature development within extra samples similarly prepared was measured for a duration of 180 min with a thermologger (Series 1000 Squirrel Meter/Logger, Eltek Ltd., Cambridge, UK) at 2.5 mm, 4 mm, and 6 mm from the surface. The thermocouples were inserted in holes drilled in the transverse side of a specimen. Two repeats were made for each density. After modification, the samples for weathering experiment were left to climatize at 20 °C, 65%RH for a few weeks, before cutting to 100 mm × 75 mm × 18 mm to facilitate placement in the artificial weathering chamber. The radial sides were sealed with one layer of silicone (Ardex GmbH, Witten, Germany) and transverse ends with a double layer. Char layer depths were determined from several cut-outs using a caliper (Table 2). Differentiating the char from the pyrolysis layer was purely visual and based on color difference between the black char and the dark brown transition layer.

### 2.1. Artificial Weathering

The artificial weathering was made with a QUV accelerated weathering tester from Q-labs using a procedure according to EN 927-6:2006 [20]. Four samples per modification were used, of which three were placed inside the chamber and one was used as unweathered reference. The weathering procedure in EN 927-6 consists of an exposure cycle of one week that is repeated 12 times (total 2016 h). The one-week cycle is made of a condensation step (24 h, 45 ℃) followed by a subcycle with UV-A (ultraviolet light-A) irradiation (2.5 h, 60 ℃) and water spray (30 min). The subcycle is repeated 48 times per one-week cycle. The spectral emission of the UV exposure is between 290–400 nm with a peak at 340 nm. The irradiance is 0.89 W/(m^2^nm) and the water spray has an effect of 6–7 L/min.

### 2.2. Evaluation of Weatherability

Visual evaluation was made according to EN 927-6. In the procedure, flaking and cracking of the surface are graded 0–5, where 0 stands for no visible defects and 5 for extensive pattern of defects. The depth of deepest crack per sample was also measured with a digital caliper from the transverse end of the sample after removing silicone. CieLAB color space was measured with Spectrolino color analyzer (X-Rite GmbH, Regensdorf, Switzerland). The variables a*, b*, L*, corresponding to red-green (scale −100–100), yellow-blue (−100–100) and lightness (0–100), respectively, were measured from unweathered references and three weathered samples from 5 points on each sample. The values from weathered samples were averaged. Total color change of ΔΕ was calculated using Equation (1):

(1) $${\Delta Ε}_{\mathrm{ab}}^{*}=\sqrt{{\left(L_{2}^{*}-L_{1}^{*}\right)}^{2}+{\left(a_{2}^{*}-a_{1}^{*}\right)}^{2}+{\left(b_{2}^{*}-b_{1}^{*}\right)}^{2}}$$

where L*_2_ − L*_1_, a*_2_ − a*_1_, b*_2_ − b*_1_ represent the differences between the original and the final coordinates (before and after weathering).

Cracking was further studied using an image processing software ImageJ v.1.50e (National Institutes of Health, Bethesda, MD, USA). The sample surface images were captured by digital camera EOS 700D (Canon, Tokyo, Japan) with lens 17–50 mm f/2.8 ex DC OS HSM (Sigma, Kawasaki, Japan) using exposure time of 1/40 s, ISO speed 100 and 50 mm focal length. The first stage of the image processing involves conversion from RGB palette with 24-bit color depth to 8-bit grayscale for the better segmentation of cracks area from the background. Identifying surface cracks involves the binary conversion of the image with manual setting of threshold parameters to identify the cracks. Once the cracks were identified on the sample surface, the calculation of cracks percentage area per total surface area was applied.

The modified (tangential) surfaces of birch and light spruce were investigated with scanning electron microscope (Zeiss Sigma VP FE-SEM (Carl Zeiss Microscopy GmbH, Jena, Germany) with acceleration voltage of 3 to 5 kV. Prior to imaging, the samples were cut to about 1 mm × 1 mm × 1 mm pieces using a razor blade, oven-dried and sputter-coated with a 5 nm layer of platinum–palladium mix.

Statistical testing was made to correlate the effect of modification, wood species, sample density (only on spruce samples), char layer thickness on cracking (area of cracking and number of cracks) using a two-sample t-test and one-way ANOVA combined with a Tukey–Kramer post-hoc test, where appropriate. Equality of variances was tested beforehand to ensure correct method.

## 3. Results

### 3.1. Temperature Logging and Char Depth

**Figure 1:**
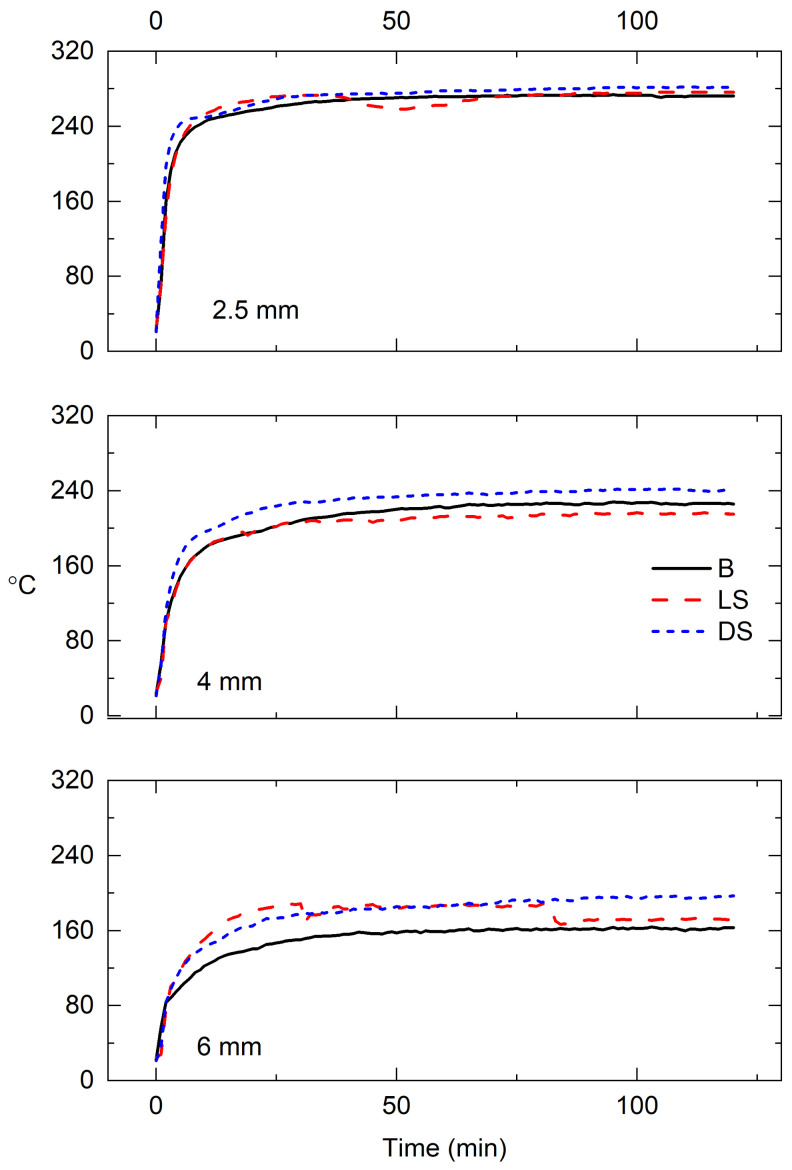
Temperature development within samples measured at 2.5, 4 and 6 mm from surface (average of two measurements per wood type). B = birch, LS = light spruce, DS = dense spruce.

The temperature development within the different wood types can be seen in Figure 1. A denser wood will conduct heat more effectively than a light wood because of its smaller internal airspace. The graph shows little differences between the wood types, but the temperature line of birch lags behind at 6 mm depth. This is most likely due to the placement of the thermocouples, as the drill hole for the wire (ø 1.2 mm) was large enough to intersect both late- and earlywood. This affects the temperature development within the measurement point, making the results somewhat directional. However, the purpose of the temperature measurements was to monitor the depth of modification. The measured char depths (Table 2) are quite well in line with the logged temperature. The formed char layer is thin and insulates the deeper layers of wood effectively, hence the temperature rises steeply during the first few minutes, after which it stalls as the char formation is complete. The temperature continues to rise very slowly within the transition zone. At 6 mm depth, the temperature is between 150 and 170 °C, which will leave the wood dehydrated and lightly thermally modified. The transition layer of birch was estimated to reach about 8 mm, so the true temperature was likely higher than that measured at 6 mm.

**Figure 2:**
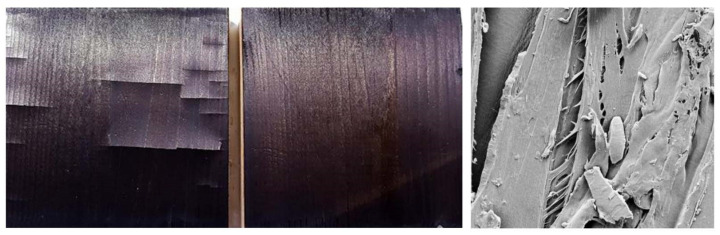
Samples DS60 with and without shallow surface checks pictured directly after modification. SEM image shows ruptured tracheid walls at 1000× magnification.

The modification temperature of 320 °C was chosen due to char line formation at about 300 °C [21], which is also the general temperature region of crystalline cellulose degradation. This ensures that the formed surface can be considered as char while keeping the temperature as low as possible to avoid extensive cracking. After preliminary testing, it was confirmed that this is the maximum temperature that can be reached using our device before severe surface cracking starts taking place. Still, some samples modified for 60 min showed shallow surface checking, and as can be seen in Figure 2, even the surface that is intact to the naked eye is cracked when investigated under the SEM. The cracking was more a rule than an exception in this most severe modification regime, but the crevices did not penetrate the entire surface layer. One of the LS60 samples cracked through the modified face, and the sample also had a resin pocket that boiled over the surface. The sample was kept for further testing to see whether the resinous surface would behave differently in weathering.

### 3.2. Visual Evaluation and Colors

**Figure 3:**
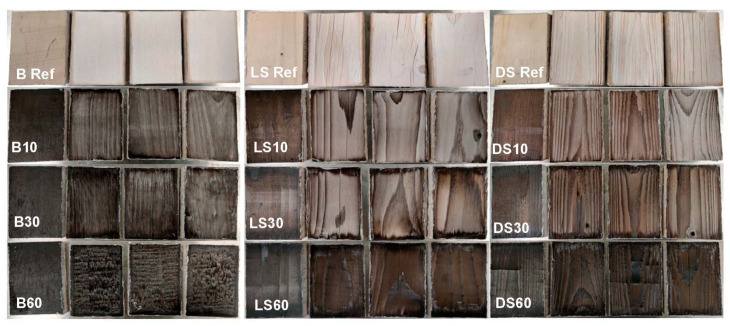
Weathered samples of birch (B), light spruce (LS), and dense spruce (DS) modified for different durations (10, 30 and 60 min) at 320 °C. The leftmost sample in the respected series is always the unweathered reference.

The artificial weathering left the samples worn and faded, but a clear trend of higher color stability in connection to the longer modification time was visible (Figure 3).

**Figure 4:**
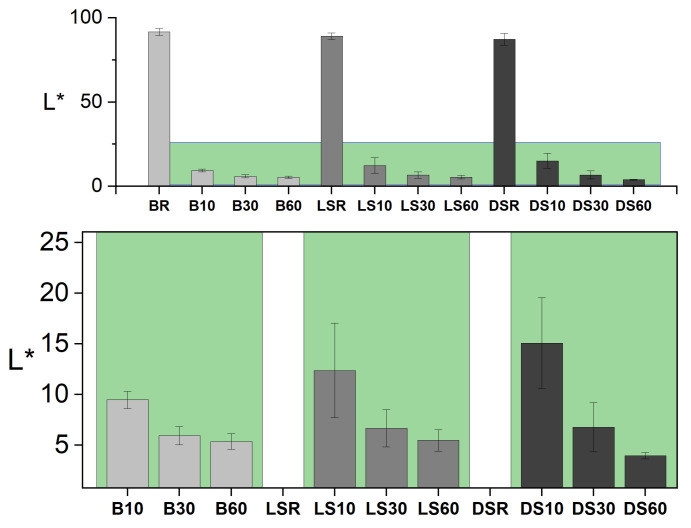
Lightness (L*) following modification. Inset (green area): modified samples.

**Figure 5:**
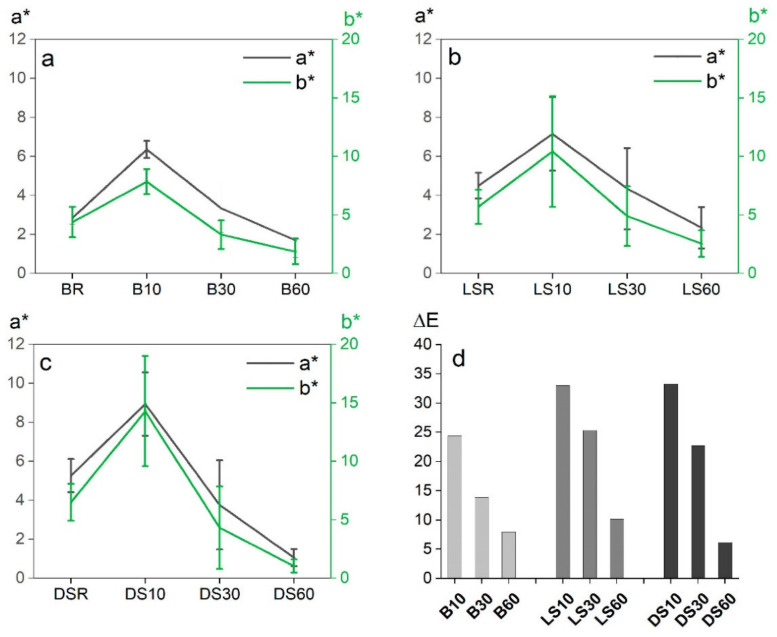
Effect of modification time on color values a* (left *y*-axis) and b* (right *y*-axis); birch (**a**); light spruce (**b**); dense spruce (**c**), measured before weathering. Graph (**d**) shows the total color change ΔΕ by species/wood type following artificial weathering.

The modification time affected the color values as expected, with a longer modification time decreasing the lightness value L* (Figure 4) and the a* and b* values (Figure 5). The differences between modifications (within species) were significant in all groups (*p* < 0.05) and according to the post-hoc test between all groups except B30 vs. B60 and LS30 vs. LS60. Between wood species, there were no significant differences. The values a* and b* translate as red-green and yellow-blue on a scale of −100 to +100. A reduction in all values is expected to take place as the surface turns more black during modification. The largest changes were seen on dense spruce where all values were more than halved. The smallest changes in color were seen on birch, where the surface was very dark already at 10 min modification (Figure 3, inside references).

The colors of the unmodified references were measured but not included in further analyses as the surfaces were lightly coated with white paint to enhance the contrast of the cracks. The color measurements of artificially weathered modified samples confirmed the visual observation of smaller ΔΕ with increasing modification time (Figure 5d). Again, the smallest overall changes are seen on the birch, indicating good color stability. Indeed, in Figure 3, the surfaces can be observed to turn black/grey in contrast to the brown/grey of the spruce surfaces. The absolute difference in ΔΕ between B60 and DS60 is very small.

### 3.3. Cracking

**Figure 6:**
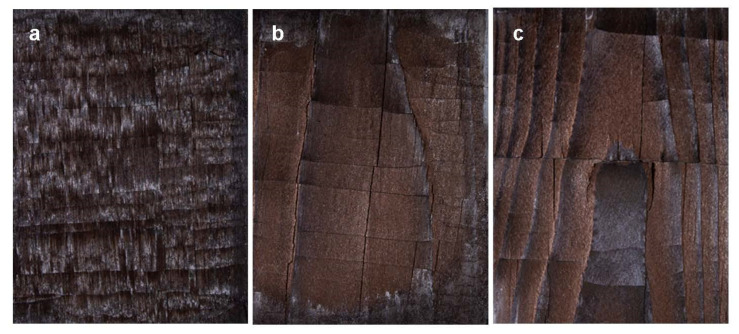
Detailed images of weathered surfaces: B60 (**a**); LS60 (**b**); DS60 (**c**).

**Figure 7:**
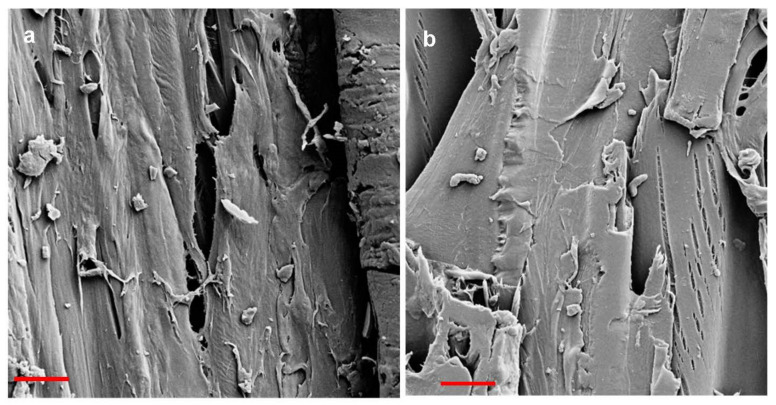
SEM images of inside stored B30 (**a**) and LS30 (**b**) surfaces showing both shrinkage and rupture. Bar = 10 µm.

**Figure 8:**
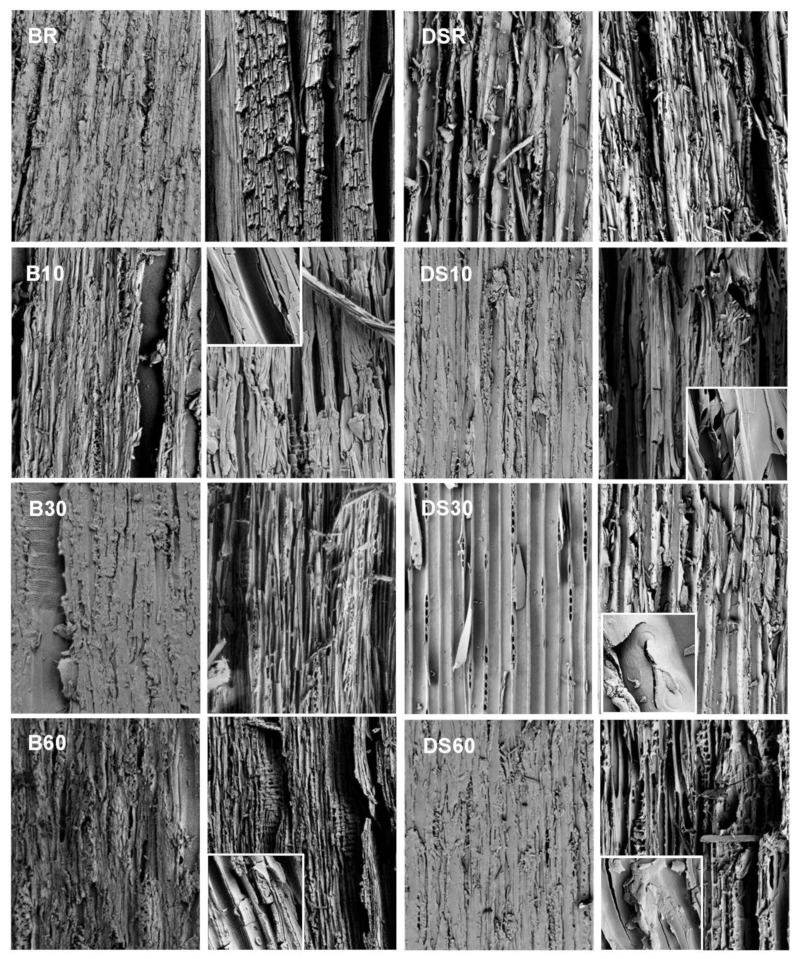
SEM images of birch (B) and dense spruce (DS) surfaces at a magnification of 100×. Left: inside stored references; right: artificially weathered specimens. Selected details in insets (at 1000–2900× magnification).

Cracking perpendicular to the fiber began between modification times of 30 and 60 mins but was missing from samples modified for 10 min. No visible cracking was present inside the stored references. All weathered samples presented longitudinal cracks ranging from less than 0.5 mm to 5.7 mm deep. The shallowest cracks were measured from BR and B60, and the deepest from LSR and LS60. The crack pattern differs between the two wood species (Figure 6). Spruce surfaces break into large rectangular pieces. The density does not seem to affect the extent of this surface checking, but it is rather specimen-specific (Figure 3). It is also somewhat dependent on cracking/checking following treatment, i.e., some of the cracks/checks existed already before modification (caused by drying and planing), and before weathering (caused by modification). The surface checking increased after more severe modification and was further increased during weathering. On birch, the crack pattern is in comparison very small-scale, although the surface similarly breaks into rectangular pieces. The thickness of these flakes was about 1 mm on LS and DS, and about 0.2 on B. In Figure 7, both wood species are seen to exhibit both shrinkage and rupture of cell walls. Shrinkage is an obvious effect of contact charring, as the modified surface cups away from the hot plate, leaving the surface in a state of compression. The SEM imaging revealed heavy microscale cracking that exhibited perpendicular fracture of fibers and erosion of plasticized surfaces (Figure 8). Deep grooves are seen in B60 after weathering, but the fibers seem to be most damaged in unmodified references. This finding is consistent with other evaluation methods. Judging by their visual appearance, B10 and DS30 are least affected by artificial weathering.

**Figure 9:**
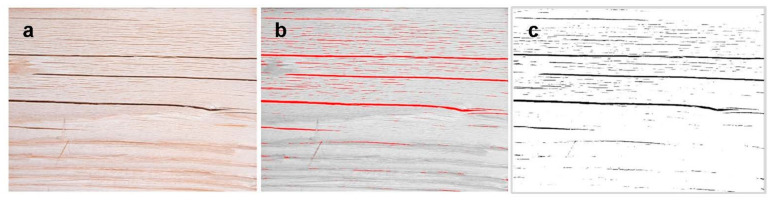
Example of image analysis using software ImageJ; captured 24-bit color depth image (**a**); 8-bit grayscale image with thresholding to identify the cracks (**b**); identified cracks for calculation (**c**).

**Figure 10:**
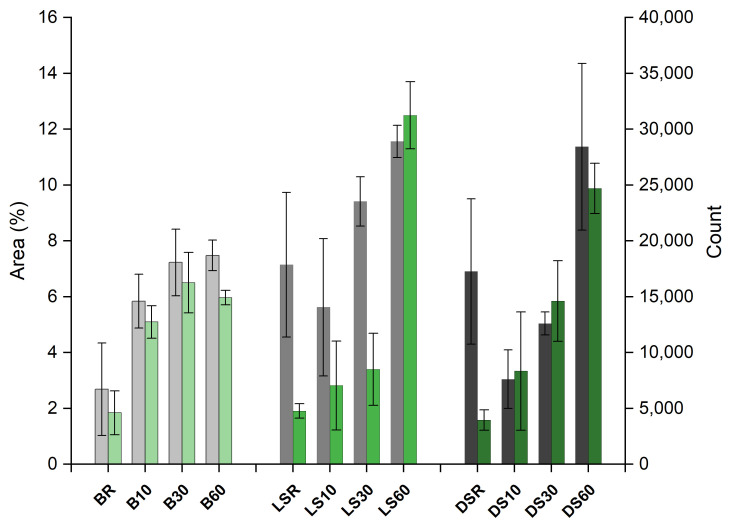
Cracked area (%, in grey) per total surface area and number of cracks (count, in green) as analyzed by ImageJ. B = birch, LS = light spruce, DS = dense spruce, R = reference; modified for 10, 30 and 60 min.

Cracking represented by ImageJ analysis of the weathered sample is depicted in Figure 9. Macroscale cracking increased with increasing modification time (Figure 10). Both the light and the dense spruce exhibited an initial decrease in the cracked area, followed by an increase. The highest cracked surface area was recorded with light spruce, although the variation between samples was highest for dense spruce. Birch showed the least increase in the cracked area between intermediate and long modification time, indicating that most of the structural changes had taken place already at the intermediate modification time of 30 min. The number of cracks (“count”) measured from both LS and DS differs slightly from the measured area, whereas with birch, both parameters are consistent.

### 3.4. The Effect of Sample Parameters on Cracking

**Figure 11:**
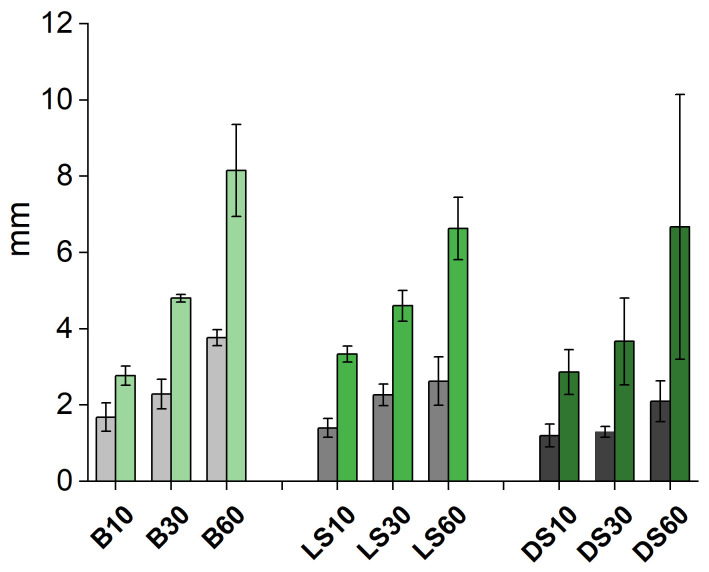
Char layer (in grey) and char and transition layer (in green) thicknesses (mm) in different modifications by species/wood type.

As expected, the area, as well as the number (“count”) of surface cracks, increased as the modification temperature increased (Figure 10). The dependence of cracking was analyzed against wood species (birch vs. light and dense spruce), modification within species/wood type, and within wood type (dense vs. light spruce), by using one-way ANOVA and two-sample t-tests. The results indicated a significant (*p* < 0.05) difference in the means of “count” between wood types at the most severe modification level (modification time of 60 min) when birch was set against dense and light spruce. The means in “area” differ significantly only between birch and dense spruce, although birch and light spruce show mild significance at *p* < 0.1. According to the post-hoc testing, differences for birch were significant in all groups except B30 vs. B60 on birch in both “count” and “area”. For DS, all groups were significantly different except DS30 vs. DS60 for “area”. Analyses for LS had non-significant results for LS10 vs. LS60 in “count”, and LSR vs. LS10 and LS10 vs. LS30 for “area”, which might indicate less of an effect with the modification, or the thinner/shorter cracks were more difficult to interpret with ImageJ. Density also seems to be a significant parameter for 60 min on “count”, whereas only for 30 min with “area”. In contrast, all groups (modifications) differ significantly on both “count” and “area”, i.e., the modification regime has a clear effect on cracking within wood species/wood types. There were no significant differences between the depth of deepest crack means except for the BR and B60. The shallowest cracks on spruce were measured from DS30, with a significant difference when compared to other DS and LS series. Measured char and transition zone thicknesses (Figure 11) were strongly correlated with the degree of cracking, both the number of and the area of the cracks (correlation coefficient = 1).

## 4. Discussion

Spruce is a favored cladding material in the Nordics because of its relatively good dimensional stability. Previous experiments with surface-charred spruce [11,12,15] have shown decreased sorption and resistance to weathering in natural test setups [10,15]. This is, in part, caused by the impermeability of spruce wood, and, in part, by thermal degradation-induced structural changes that reduce adsorption, discoloration by mold fungi and susceptibility to photodegradation. The porous structure of char absorbs liquid water readily, but contact charring creates a surface that is compacted, much harder and smoother. The process is dependent on sufficient contact that ensures even modification of the entire surface. If the wood is not oven-dried, sudden exposure to a high temperature will cup the wood surface away from the heated plate, which leaves the center less modified than the edges. Therefore, a small weight was used to ensure the connection between the wood surface and the hot plate but avoid excessive cellular damage. For larger scale specimens such as cladding boards, a finetuning of pressure would be needed to facilitate contact charring so that the surface stays flat during modification but keeps surface stresses minimal. A continuous contact charring device was introduced in [15], but inadequate surface pressure left the char layer very thin and flaky.

Unlike spruce, birch is generally not used in exterior applications because of its tendency to mold. However, the high-temperature thermal modification also allows outside use [22]. Recently, Kymäläinen et al. [10] reported rather good weatherability of charred birch, although the contact-charred samples (equivalent to B30 in this study) suffered from fading of color. The color stability (evaluated by the magnitude of changes in L* and ΔΕ) was however better than on spruce and pine. Here, in terms of color, the birch samples performed well in comparison to spruce. Hardwoods are more susceptible to thermal degradation, and a darker surface formed within a shorter modification time. The color stability in weathering was best for birch at modification times of 10 min and 30 min, but the ΔΕ of B60 was of the same magnitude as for light spruce, and finally, the color stability was highest for dense spruce modified for 60 min. However, the surface of B60 was very black to begin with, in comparison to the black-brown of both dense and light spruce. If the ΔΕ was to be compared with a painted surface, the 60 min samples would fall somewhere in the middle of the artificially weathered paint systems evaluated by Van den Bulcke et al. [23], corresponding to semi-transparent solvent-borne alkyd coatings, with opaque solvent-borne alkyds performing better and water-borne acrylate and polyurethane systems performing worse. The ΔΕ of 60 min samples is also closing in on the value for flame-charred surfaces recorded by Kymäläinen et al. [10] in a natural weathering setup. In the said study, the color stability of flame-charred surfaces was comparable to a double layer of an opaque black water-borne alkyd coating on primer paint, with ΔΕ values below two.

The crack pattern of birch and spruce is very different: birch surface experiences more small-dimension flaking, whereas spruce surfaces undergo checking with larger pieces partly breaking off, as seen in Figure 6. Although the birch surfaces showed fewer large cracks at the most severe modification regime (Figure 10), the small-scale checking combined with flaking leads to clear washed-out regions where whitish colors are revealed, partly explaining the higher ΔΕ of B60. This flaking surface char is very thin in comparison to the pieces that are partly breaking off from the spruce surfaces. Additionally, from the microscope images, it was seen that although the surface is hard and smooth, there is extensive microscale cracking that in practice increases the surface area available for moisture transport. Moisture content was not measured during the weathering experiment, but it is connected to cracking and therefore to the weatherability of the wood in use. Continuous shrinking/swelling stresses create microcracks that create paths for moisture to reach the more hydrophilic parts of the wood. For example, Sjökvist et al. [9] noted that high-density spruce samples cracked more in natural weathering, and as a result, absorbed more moisture than their lighter counterparts. The length, width and depth of cracks on the coating as well as the wood surface had a clear effect on the increase in moisture content.

Despite using a weight on top of the sample during modification there was some cupping effect, most visible in DS where measured char thicknesses have the highest SD (Figure 11). Birch has a higher density, but the variation in measured char layer thicknesses was much lower than on DS—the cupping distortion was, therefore, smaller for birch than for dense spruce. Density, however, is not a very good estimator of drying distortion [24] that is the dominant reaction in heat exposure. The grain direction, especially the existence of spiral grain as well as the presence of juvenile wood strongly affects the level of distortion [25]. The microfibril angle (MFA) is another important indicator, as its increase results in a drastic increase in longitudinal shrinkage [26]. Here, the existence of juvenile wood may be eliminated because the wood samples were extracted from sapwood, but it is possible the grain direction deviated from straight within the samples, although care was taken to ensure reproducibility. Unusual MFA may also be disregarded as no unusual shrinkage was observed in modification (tangential shrinkage) nor weathering (elongation in wetting causing longitudinal and tangential cracking). Density also has a rather minor effect on the one-dimensional charring rate, which has in practical settings been agreed as 0.5 mm/min for hardwoods and 0.65 mm/min for softwoods [21]. Several parameters have an effect, e.g., softwoods, in particular, may char in a more unpredictable manner mainly because of resin pockets that increase flame spread rate [27]. Resin pockets were here excluded except from one sample, which did not exhibit any differences in comparison to other samples in the group, but the colors faded similarly. This pocket was not visible but burst during modification, and it is likely that in other samples, there may have been smaller or deeper situated ducts that would affect the final charring behavior. In the literature, the role of extractives in thermal modification results is unclear [28,29]. In the temperature development graph (Figure 1), minor differences can be seen at measuring points further from the surface, although unfortunately, the between-samples variation caused some inconsistencies. From char layer thickness measurements, a difference is seen between LS and DS at 60 min, where it seems that denser, more thermally conductive wood overrides the hindering effect of char layer formation leading to a higher temperature at a deeper location within the wood. The heat penetration is aided by the cracking of the surface char.

The exposed wood face affects the cracking behavior: tangential surfaces, in particular, may experience diagonal microchecking through bordered pits (and loss of ray cell walls, with checking following the fibril angle on the S2 wall layers (reported for uncoated, unmodified redwood [30])). In carbonized wood, rays split and crack more with increasing temperatures [31,32]. With thermally modified wood exposed to weathering, a higher modification temperature (190 vs. 220 °C) produced more severe weathering erosion of the secondary wall [18]. This is due to a higher lignin content in S2 that causes a higher degree of erosion during weathering as lignin is the primary target for photodegradation. Longitudinal microcracks, caused by shrinkage and swelling in tracheids, were also seen, and plasticization appeared to increase cracking. The authors, therefore, stated that thermal modification is beneficial for aesthetic properties, but harmful for structural properties [18]. Similar longitudinal cracks are seen in Figure 7 and Figure 8 but also fracture of fibers and severe erosion grooves. Erosion of pits and especially of birch ray parenchyma is obvious, as well as the dilation of ray spaces. The depth of cracks was not measured, and it is not clear whether they become deeper with more severe modification, but they do seem comparable in size to those in weathered unmodified references. The crack area analysis of DS10 and DS30 present the least visible cracks. LS10 also shows a decrease in the cracked surface area in comparison to other modifications. The LS10 was also not significantly different from LSR and LS30 on “area”, nor from LS60 on “count”. This may be caused by the wood density, or the shape and size of the cracks were more difficult to decipher with the image analysis program. At a magnification of 100× (Figure 8), DS10 (and B10) show a plasticized surface with less deep erosion grooves than in other modification regimes—DS30 is also rather intact in comparison, revealing a connection between visible and microscopic cracks. However, with more in-depth scrutiny, cracks measuring less than a micrometer (inset in Figure 8 DS30), as well as heavily degraded pit structures (Figure 8 DS10), can be detected. The char cracking is a result of a strong restriction of shrinkage parallel to the grain, where only shallow fissures are produced [33]. The higher modification temperature leads to cuts through the wood fibers, developing deep into the sample. As this is an inherent property of wood, it may be impossible to contact char the wood surface sufficiently without breaking it at least to a degree. Plasticizing the surface further with additives could in theory change the outcome but would likely also increase the related costs, as well as change the pyrolysis behavior of the wood. Earlier research has shown that a higher modification temperature induced by flame (surface temperature exceeding 1000 °C) creates a recalcitrant aromatic char that withstands weathering stresses well [10], whereas an FT–IR analysis revealed minor damage to wood contact-charred at about 450 °C [15]. Similar properties can be obtained by increasing modification temperature or the modification time [11,34]. This is clearly seen in the improving color stability of samples modified for 60 min. However, to produce a surface that could be used in exterior applications, even higher modification temperatures should be used despite the more extensive cracking of the surface. A shorter modification time in connection to higher temperatures would also be the more economical option. For aesthetic reasons, it might be beneficial to apply a shorter modification time/lower modification temperature for a less cracked surface, but concluding from these results, this sort of a product would only be suitable for indoor use.

## 5. Conclusions

This study evaluated the surface properties of contact-charred birch and spruce at variable modification times at a set temperature to elucidate the durability in use as an exterior cladding material. Surface cracking evaluated by area was significantly dependent on modification time and, in connection, char layer. There was little connection between the cracked surface area and the density/wood species, but the total number of cracks depended on these parameters. Color stability also improved with a longer modification time. The cracking pattern of birch and spruce was very different, and despite some flaking of the surface char layer, birch exhibited rather a good color stability after artificial weathering. The overall color change ΔΕ was smaller than those recorded for the two types of spruce wood at shorter modification times. In terms of weathering resistance, dense spruce performed best at the highest modification time. The ΔΕ was also by far the smallest. Considering the high cracked surface area, the less severe modification conditions were better for this material, but it remains to be investigated whether the surface cracking is actually detrimental to the wood’s performance in practice, i.e., how deep the formed crevices are, and whether they are large enough to allow water molecule ingress. The ultrastructural changes after artificial weathering were also strongly connected to the modification time. In comparison to unmodified references, less fractured fibers were seen on birch and narrower erosion grooves between tracheids on spruce at certain modification regimes. In terms of producing a weather-resistant contact-charred façade, even higher temperatures may be needed despite more extensive cracking. The weathering resistance is therefore likely to be more connected to the increasing chemical inertness of the material (i.e., more carbonized char) than the actual mechanical performance of the cracked surface.

## Figures and Tables

**Table 1:** Time–temperature regimes and sample groups, standard deviation in parenthesis.

	Birch	Light Spruce	Dense Spruce
**Density [kg/cm^3^]**	0.622 (0.02)	0.352 (0.02)	0.437 (0.04)
**Modification time [min]**	0 10 30 60	0 10 30 60	0 10 30 60
**Coding**	BR B10 B30 B60	LSR LS10 LS30 LS60	DSR DS10 DS30 DS60

**Table 2:** Char and transition layer thicknesses are measured from cut-outs of several samples, standard deviation in parenthesis.

	Birch			Spruce, Light			Spruce, Dense		
**Coding**	**B10**	**B30**	**B60**	**LS10**	**LS30**	**LS60**	**DS10**	**DS30**	**DS60**
**Char thickness [mm]**	1.7 (0.4)	2.3 (0.4)	3.8 (0.2)	1.4 (0.2)	2.3 (0.3)	2.6 (0.6)	1.2 (0.3)	1.3 (0.1)	2.1 (0.5)
**Char + transition layer [mm]**	2.8 (0.3)	4.8 (0.0)	8.2 (1.2)	3.3 (0.2)	4.6 (0.4)	6.6 (0.8)	2.9 (0.6)	3.7 (1.1)	6.7 (3.5)

## Data Availability

The data presented in this study are available on request from the corresponding author. The data are not publicly available due to patenting interests.
